# Hyperpolarized ^129^Xe MR Imaging of Alveolar Gas Uptake in Humans

**DOI:** 10.1371/journal.pone.0012192

**Published:** 2010-08-16

**Authors:** Zackary I. Cleveland, Gary P. Cofer, Gregory Metz, Denise Beaver, John Nouls, S. Sivaram Kaushik, Monica Kraft, Jan Wolber, Kevin T. Kelly, H. Page McAdams, Bastiaan Driehuys

**Affiliations:** 1 Center for In Vivo Microscopy, Duke University Medical Center, Durham, North Carolina, United States of America; 2 Department of Radiology, Duke University Medical Center, Durham, North Carolina, United States of America; 3 Department of Medicine, Duke University Medical Center, Durham, North Carolina, United States of America; 4 GE Healthcare, Amersham, United Kingdom; 5 Department of Radiation Oncology, Duke University Medical Center, Durham, North Carolina, United States of America; University of Illinois at Chicago, United States of America

## Abstract

**Background:**

One of the central physiological functions of the lungs is to transfer inhaled gases from the alveoli to pulmonary capillary blood. However, current measures of alveolar gas uptake provide only global information and thus lack the sensitivity and specificity needed to account for regional variations in gas exchange.

**Methods and Principal Findings:**

Here we exploit the solubility, high magnetic resonance (MR) signal intensity, and large chemical shift of hyperpolarized (HP) ^129^Xe to probe the regional uptake of alveolar gases by directly imaging HP ^129^Xe dissolved in the gas exchange tissues and pulmonary capillary blood of human subjects. The resulting single breath-hold, three-dimensional MR images are optimized using millisecond repetition times and high flip angle radio-frequency pulses, because the dissolved HP ^129^Xe magnetization is rapidly replenished by diffusive exchange with alveolar ^129^Xe. The dissolved HP ^129^Xe MR images display significant, directional heterogeneity, with increased signal intensity observed from the gravity-dependent portions of the lungs.

**Conclusions:**

The features observed in dissolved-phase ^129^Xe MR images are consistent with gravity-dependent lung deformation, which produces increased ventilation, reduced alveolar size (i.e., higher surface-to-volume ratios), higher tissue densities, and increased perfusion in the dependent portions of the lungs. Thus, these results suggest that dissolved HP ^129^Xe imaging reports on pulmonary function at a fundamental level.

## Introduction

Enabling the diffusive exchange of alveolar gases with pulmonary blood is the most fundamental physiological function of the lungs. The uptake of alveolar gases, referred to as the diffusing capacity or conductance, consists of two serially ordered components [1]. The first, in which gases diffuse through a semi-solid membrane comprising the alveolar epithelium and capillary walls, is determined primarily by the thickness and surface area of the membrane and is referred to as the ‘membrane diffusing capacity’ of the lungs. The second component, which is applicable to hemoglobin-binding gases such as O_2_ and CO, is referred to as the ‘reactive conductance’ and depends on the reaction rate with the blood and the capillary blood volume. Diffusing capacity is most commonly measured using carbon monoxide (DL_CO_), which is currently the primary means of directly assessing normal gas uptake and diagnosing pathological changes in gas exchange that occur in such disorders as interstitial lung disease and chronic obstructive pulmonary disease [2], [3]. Unfortunately, DL_CO_ is only a global measure of gas uptake and, thus, cannot provide information about normal spatial variations in gas exchange or, more importantly, heterogeneity caused by disease.

Alternatively, diffusing capacity can be estimated using stereological methods, which provide regional information at microscopic resolution, applied to fixed lung tissues [4], [5]. However, these morphometric approaches cannot yield whole-lung information *in vivo*. Furthermore, estimates of diffusing capacity and capillary blood volume obtained through stereological and DL_CO_-based measurements differ by a factor of two or more [6], [7]. Because of these inherent limitations in the commonly used methods, a significant need exists to develop noninvasive and spatially resolved methods to measure the diffusive uptake of alveolar gases by the lungs. A particularly promising candidate for providing clarifying information is hyperpolarized (HP) ^129^Xe, which is a gaseous magnetic resonance imaging (MRI) contrast agent that generates high, nonequilibrium signal intensity [8], and which can be used to image the pulmonary airspaces of human subjects [9], [10], [11].

HP ^129^Xe displays a reasonably high Ostwald solubility in many tissues [12], including the blood (∼14%), and exhibits an unusually large, environmentally dependent nuclear magnetic resonance (NMR) chemical shift range (>200 ppm *in vivo*) that allows dissolved ^129^Xe to be excited and detected separately from gas-phase ^129^Xe [8]. Although xenon possesses anesthetic properties at sufficiently high partial pressures [13], it is chemically inert and not normally present at detectable levels in human lungs, making HP ^129^Xe a potentially powerful, noninvasive probe of gas exchange. These properties have already been exploited in animals to globally assess pulmonary gas exchange capacity [14], [15], [16] and interstitial membrane thickness [16] using NMR spectroscopy. HP ^129^Xe NMR spectroscopy has also been used to estimate surface-to-volume ratios in the lungs of healthy human subjects [10]. However, harnessing these unique MR properties to examine regional pulmonary gas exchange is challenging, because the low density of lung tissues, moderate solubility of xenon, and unfavorable T_2__*_ of dissolved HP ^129^Xe (1.5–2.4 ms at 1.5 T as determined by whole-lung spectroscopy) generate signals that are only a few percent as large as those from HP ^129^Xe in the pulmonary airspaces.

Despite the intrinsically low signal intensity, direct imaging of HP ^129^Xe dissolved in pulmonary tissues has been reported in rats using chemical shift imaging [17]. However, these images required many breaths of ^129^Xe and provided only non-slice selective, one-dimensional (1D) and low-resolution, 2D (matrix = 16×16; pixel size = 14.1×14.1 to 39.1×39.1 mm^2^) images of the entire animal. To avoid the low dissolved-phase signal and to generate higher-resolution images, the spatial distribution of ^129^Xe exchange has most commonly been probed indirectly through Xenon polarization Transfer Contrast (XTC) imaging [11], [18], [19], [20], in which dissolved HP ^129^Xe magnetization is intentionally saturated and diffusive exchange between the pulmonary tissues and alveolar spaces is exploited to attenuate the gas-phase ^129^Xe signal intensity.

More recently, it was shown that 2D radial MR imaging could overcome the short dissolved-phase T_2__*_ and directly image ^129^Xe in the pulmonary tissues of rats [21] at relatively high resolution (matrix = 64×64; pixel size = 1.25×1.25 mm^2^). In that work, however, the other factors that contribute to low dissolved ^129^Xe intensity were compensated for by signal averaging over 40 or more breaths, which required the use of a constant volume ventilator capable of mixing the HP gas with oxygen. This technical burden, coupled with the hour-long polarization times required to generate liter-volumes of HP ^129^Xe and the possible cumulative buildup of anesthetic xenon gas, make multiple-breath signal averaging impractical in humans. Thus, it has been unclear whether meaningful images of dissolved ^129^Xe could be obtained from human subjects.

In this paper, we demonstrate the feasibility of generating three-dimensional (3D) images of HP ^129^Xe dissolved in the pulmonary tissues of human subjects during a single, 16-second breath-hold. Additionally, we investigate the optimal imaging parameters needed to generate dissolved ^129^Xe images. Finally, the optimal imaging parameters and the signal intensity patterns observed from the images are related to the physical processes of pulmonary gas transport and to known pulmonary physiology.

## Materials and Methods

### Ethics Statement

Experiments were conducted under GE Healthcare's Investigational New Drug application for HP ^129^Xe MRI and were approved by the Duke University Medical Center Institutional Review Board. All subjects provided written, informed consent prior to imaging.

### ^129^Xe Polarization and Delivery

1.0-L doses of isotopically enriched xenon (83% ^129^Xe, Spectra Gases Inc., Alpha, NJ) were hyperpolarized by rubidium vapor spin exchange optical pumping, cryogenically accumulated [22] using a prototype polarizer (GE Healthcare, Durham, NC), and thawed into Tedlar bags (Jensen Inert Products, Coral Springs, FL). The ^129^Xe polarization, which was typically 5–9% after accumulation and thawing, was measured with a prototype polarization measurement station (GE Healthcare). Prior to experiments, subjects were instructed to exhale to functional residual capacity before inhaling the entire 1-L volume of HP gas. Subjects then held their breath for the duration of the MR data acquisition (<16 seconds). No subject received more than 4 total doses of HP ^129^Xe.

### MR Spectroscopy and Imaging

MR imaging and spectroscopy were performed using a 1.5-T whole-body MRI scanner (EXCITE 14M5; GE Healthcare, Milwaukee, WI). Subjects were fitted with a 17.66 MHz quadrature vest coil (Clinical MR Solutions, Brookfield, WI) that was proton-blocked to permit shimming and localizing with the scanner's ^1^H body coil. HP ^129^Xe ventilation images [field of view (FOV) = 40×40 cm^2^, slice thickness = 15 mm, TR = 7.9 ms, echo time (TE) = 1.9 ms, bandwidth (BW) = 8.0 kHz, and flip angle (α) = 5–7°] were acquired using a slice-selective spoiled gradient recalled echo (SPGRE) sequence. Slices were acquired in the anterior-to-posterior direction. All ventilation images were acquired with 128 frequency-encoding points, but the number of phase-encoding steps was varied from 90 to 128 depending on lung size. Dissolved ^129^Xe images were acquired using a 3D radial sequence that employed a pseudo-random view ordering [23], [24] and that selectively excited HP ^129^Xe with a 1.2-ms, 3-lobe sinc pulse applied at a frequency 3826 Hz higher than the gas phase ^129^Xe resonance. Unless otherwise stated, each dissolved image comprised 3751 radial views (α = 8°, matrix = 32^3^, FOV = 40×40×48 cm^3^, TR/TE = 4.2/0.9 ms, BW = 15.6 kHz.)

Following acquisition, radial images were reconstructed offline using non-uniform fast Fourier transform (NUFFT). Briefly, NUFFT reconstruction employs a least squares optimized kernel to interpolate the nonuniform, radial k-space data onto a uniform grid prior to Fourier transform. For more detail, see ref. [24]. Dissolved ^129^Xe images were overlaid on ^129^Xe ventilation MR images in OsiriX (OsiriX Foundation, Genève, Switzerland). SNR measurements were made by selecting regions of interest in ImageJ (U. S. National Institutes of Health, Bethesda, MD, http://rsb.info.nih.gov/ij/). The signal intensity from radial k-space rays (i.e. the magnitude of the data point at k = 0) was extracted from the raw image data using routines written in MATLAB (The MathWorks, Inc., Natick, MA). Spectra were processed using HiRes 1.6 (Hatch Center for MR Research, Columbia University, New York, NY).

### Subject Selection and HP ^129^Xe Dosing

Subjects were enrolled in the study from a population of healthy volunteers (19 to 57 years of age) who had not smoked for at least 5 years and had a smoking history of less than 5 pack-years. While in the MR scanner, all subjects had their blood pressure, heart rate, and oxygen saturation level measured using a Datex-Ohmeda monitoring system (GE Healthcare, Helsinki, Finland). Each subject received one dose of HP ^129^Xe for ventilation imaging. Five subjects underwent spectroscopy experiments to establish the consistency of the dissolved ^129^Xe resonance frequencies. Five additional subjects were imaged in studies in which the flip angle was varied from α≈3° to 17° to optimize the dissolved HP ^129^Xe MR imaging strategy (for the parameters stated earlier). Three additional subjects (for a total of four subjects) were imaged using the optimized flip angle (α≈8°). Spectroscopy, ventilation imaging, and dissolved ^129^Xe imaging were performed in the supine position for all 14 subjects. For one individual, a dissolved ^129^Xe image was also obtained in the prone position.

## Results and Discussion

### HP ^129^Xe Spectral Characteristics and Magnetization Dynamics

**Figure 1:**
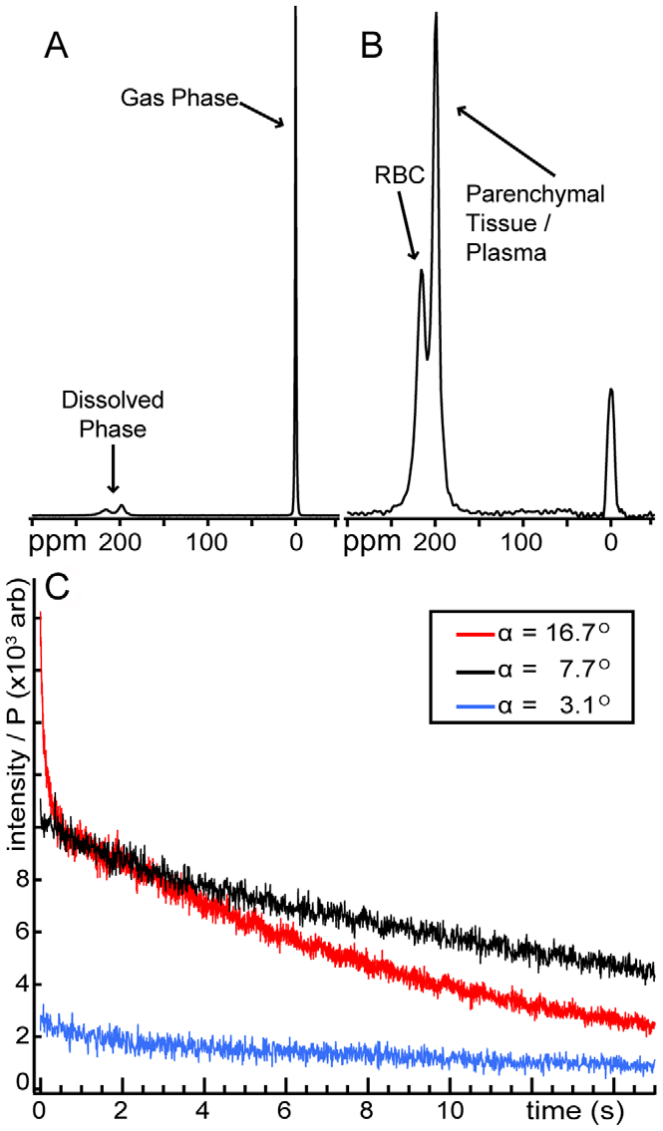
HP ^129^Xe MR signal intensity in human lungs. (**A**) NMR spectrum obtained using a hard, 7° RF pulse. The gaseous HP ^129^Xe signal is used as a 0 ppm reference. (**B**) Spectrum from a selective, 7° pulse centered at 218 ppm. The 218-ppm peak arises from ^129^Xe dissolved in the red blood cells (RBC), and the 197-ppm arises from ^129^Xe in the blood plasma and semi-solid parenchymal tissues. (**C**) Dissolved HP ^129^Xe signal dynamics during single breath-hold radial imaging. Data points represent the magnitude of k-zero from each radial view weighted by the initial HP ^129^Xe polarization (P). Even using a relatively large flip angle of α∼17° and a rapid TR of 4.2 ms, substantial dissolved signal is still observed at the end of the breath-hold period due to rapid, diffusive replenishment of dissolved ^129^Xe magnetization.

As was observed previously in humans [9], the ^129^Xe NMR spectrum displays three distinct resonances immediately after inhalation, (see Figure 1A and 1B). The lowest frequency peak (used as the 0 ppm reference) originates from gaseous xenon in the major airways, bronchioles, and the alveolar spaces. Two broad, partially overlapping peaks, collectively representing the dissolved phase, are also observed at 197 ppm and 218 ppm and arise from HP ^129^Xe dissolved in the blood plasma and parenchymal tissues and ^129^Xe dissolved in the red blood cells (RBCs), respectively [25]. For all five individuals in the spectroscopy studies, the resonant frequencies of the plasma/parenchyma and RBC peaks were found to be relatively constant at 197.3±0.5 and 218.3±1.0 ppm, respectively.

The spectrum in Figure 1A was acquired using a hard radio-frequency (RF) pulse with a flip angle of ∼7° and shows that the dissolved compartment contributes a rather small signal compared to gas-phase ^129^Xe. However, Figure 1B demonstrates that the dissolved-phase ^129^Xe resonances can be excited largely to the exclusion of the gas-phase resonance using a 1.2-ms, 3-lobe sinc pulse centered at 218 ppm. Such selective excitation is crucial for imaging the dissolved phase, while preserving the gas-phase magnetization. The residual gas-phase signal could not be completely eliminated by increasing the length, and thus the selectivity, of the RF pulse, most likely due to hardware limitations.

In conventional MR imaging of HP gases in the alveolar spaces, a large but finite amount of non-equilibrium magnetization is inhaled. This magnetization is then reduced by longitudinal relaxation and, more importantly for MRI, consumed by every RF pulse applied during image acquisition [26]. Due to its solubility, a small amount of the HP ^129^Xe magnetization is also lost to capillary blood flow away from the alveolar regions [16], [17], [26]. Therefore, HP gas images are typically acquired using small flip angles to conserve the non-renewable longitudinal magnetization and to minimize k-space blurring due to the ever decreasing levels of transverse magnetization [27]. While signal attenuation remains a concern for dissolved HP ^129^Xe MR imaging, and the low dissolved intensity demonstrated in Figure 1B represents a significant challenge, the difficulties are partially overcome by the physical nature of the lung itself, which promotes highly efficient gas exchange.

Like gas-phase HP ^129^Xe, dissolved ^129^Xe magnetization is subject to losses caused by relaxation, blood flow, and RF. However, the dissolved magnetization is continuously replenished by HP ^129^Xe diffusing in from the alveolar compartment. In healthy individuals, this diffusive replenishment is remarkably rapid because the thin alveolar septum (septal thickness, 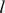
<10 µm [4]) and moderate diffusion coefficient of ^129^Xe dissolved in the pulmonary tissues (*D* = 0.33×10^−5^ cm^2^ s^−1^ [19]) combine to yield a timescale for complete exchange (i.e., ∼*l^2^/2D*) of ∼100 ms. These rapid dissolved ^129^Xe replenishment times, which have been verified spectroscopically [11], [15], [16], [21], produce a *T_1_*-like magnetization recovery in the gas-exchange tissues that enable the use of relatively large flip angles and fast repetition times (TR) to overcome the small instantaneous signals.

The effect of rapid dissolved-phase ^129^Xe replenishment is demonstrated in Figure 1C, which shows the magnitude of the k-zero intensity (i.e., the first data point in each radial view, which is acquired in the absence of imaging gradients and, thus, represents the total signal intensity) from three subjects. These dissolved-phase data were acquired with TR = 4.2 ms and three different flip angles. The smallest flip angle (∼3°) produced a small, but relatively uniform, signal intensity profile that decayed by only 49% during the 16-second acquisition. This low level of attenuation indicates that the dissolved and gas-phase ^129^Xe reservoirs are in rapid exchange on the imaging timescale, because in the absence of diffusive replenishment, the 3° RF pulses would have caused a 50% signal decay in only 2 seconds. Moreover, the observed signal loss can be largely attributed to gas-phase ^129^Xe relaxation caused by dipole coupling to paramagnetic O_2_ gas [28] in the alveolar spaces. For instance, even a relatively small O_2_ partial pressure of 0.1 bar (10 kPa) would produce a ^129^Xe T_1_ of 27 seconds and reduce the available magnetization by 45% during a 16-second image acquisition.

In contrast to the 3° flip angle, the relatively high (16.7°) flip angle generated larger signal intensities but produced substantially faster signal decay. This more rapid signal attenuation, which is conceptually similar to the contrast generating mechanism in XTC imaging [18], is due to RF-depolarized ^129^Xe continuously returning to the alveolar spaces and depleting the gas-phase magnetization reservoir. Although the larger flip angle generated a higher level of indirect RF depletion, 13% of the initial dissolved signal still remained at the end of the breath-hold period (i.e., after applying 3751 RF pulses). If diffusive gas exchange did not play a dominant role in the dissolved HP ^129^Xe magnetization dynamics, this level of signal intensity loss would be reached in only 200 ms using just 47 RF pulses.

The intermediate flip angle (7.7°) provides a reasonable compromise between the need to obtain high signal intensities and maintain uniform signals during the entire scan. We empirically found that, for TR = 4.2 ms, flip angles near 8° provided the highest-quality dissolved HP ^129^Xe images. More rigorous optimization will require a model that takes into account all sources of signal attenuation and the diffusive exchange of xenon between lung compartments.

### 3D MR imaging of dissolved HP ^129^Xe

**Figure 2:**
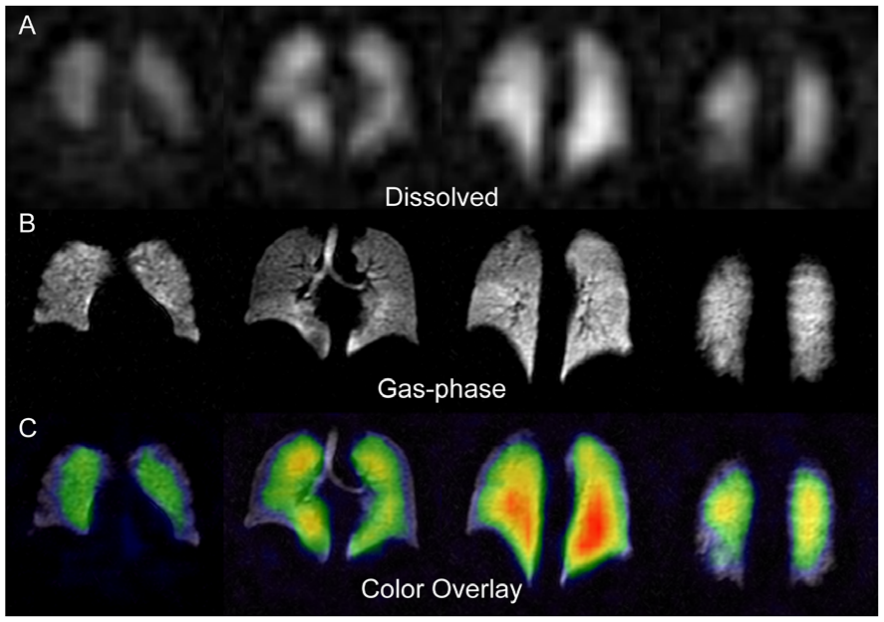
HP ^129^Xe MR imaging. Panels are arranged with the more anterior portions of the lungs shown to the left and posterior portions to the right. (**A**) 15-mm-thick sections from a dissolved-phase HP ^129^Xe image (12.5×12.5 mm^2^ in-plane resolution) of a healthy human volunteer. (**B**) Corresponding 15-mm-thick slices from a gas-phase HP ^129^Xe image of the same subject (3.2×3.2 mm^2^ in-plane resolution). (**C**) Dissolved ^129^Xe image from (A) displayed in color and overlaid on the grayscale ventilation image from (B).

Figure 2A shows 15-mm-thick sections from an optimized (i.e., α = 8°) dissolved HP ^129^Xe image of a healthy human volunteer. This 3D image was acquired with a resolution of 12.5×12.5×15 mm^3^ in less than 16 seconds. Despite lower resolution, the dissolved image exhibits the same overall shape as the corresponding gas-phase image (Figure 2B), indicating that gas uptake occurred throughout the ventilated portions of the lungs. An exception to the general agreement between dissolved and ventilation images is observed in the regions of the lungs corresponding to the major airways, which are clearly visible in the ventilation images in Figure 2B. In contrast, Figure 2A and all other dissolved HP ^129^Xe images exhibit no signal from these regions, indicating that dissolved signal can only be observed from portions of the lungs that are actively involved in gas exchange. A similar agreement between the spatial composition of dissolved images and the expected physiology was observed previously in 2D imaging studies of rats [21].

The 3D character of these dissolved images, however, allows additional features to be visualized. Specifically, substantial anterior-to-posterior differences in signal intensity are observed in the lungs. These differences are better appreciated in Figure 2C, which displays the dissolved image overlaid in color on the grayscale ventilation image. Anterior-to-posterior variations in the dissolved ^129^Xe signal intensity were observed from all subjects. As will be explained in subsequent sections, much of this directional variation in SNR likely arises from physiological variations in the lungs themselves.

Additionally, Figure 2 also demonstrates a degree of in-plane heterogeneity, with reduced signal being observed from the periphery of the lungs. This heterogeneity most likely does not reflect an underlying heterogeneity in gas-exchange, but rather, a reduced ventilation in the periphery of the lungs [29]. Alternately, this iso-gravitational heterogeneity may be an artifact resulting from the modest overall SNR of the dissolved images. Subject-to-subject variations in signal intensity (whole-lung SNR = 6.5 to 11.0) were also observed due to differing levels of ^129^Xe polarization and possibly to differences in the subjects' lung volume.

### Off-Resonant Excitation of Gas-Phase HP ^129^Xe

**Figure 3:**
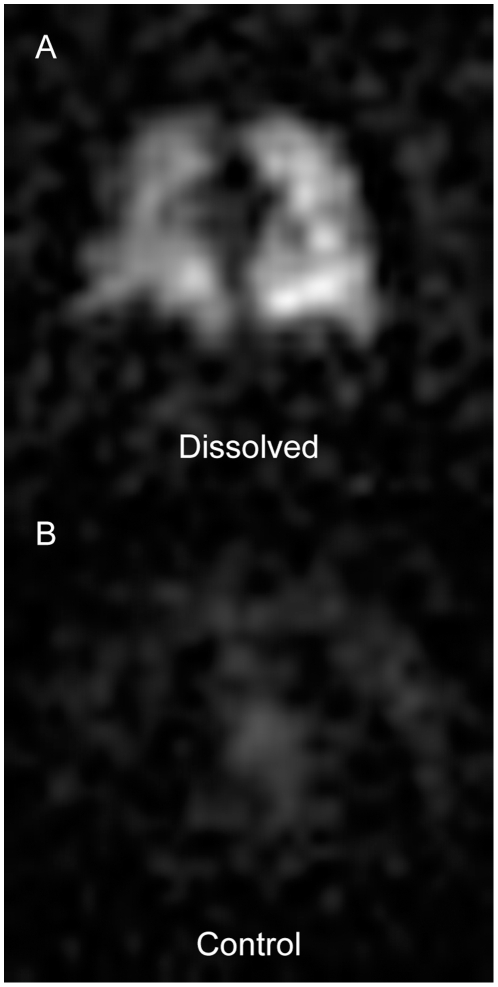
Off-resonant excitation of gas-phase ^129^Xe magnetization. (**A**) Representative 15-mm-thick section from a standard dissolved HP ^129^Xe image (α = 8°, RF centered 3826 Hz above the gas-phase resonance) of a supine subject. (**B**) Corresponding 15-mm-thick section from a control image of the same supine subject. The MRI acquisition parameters were identical to those used to produce the image in (A) except that the RF pulse was centered 3826 Hz below the gas-phase resonance. Windowing and leveling were identical for both (A) and (B).

Before considering the physiological sources of dissolved ^129^Xe image heterogeneity, one potential source of image artifacts must be discussed. Specifically, signal contamination caused by unintentionally excited gas-phase ^129^Xe magnetization (see Figure 1B) could also contribute to variations in image intensity. The effects of off-resonant excitation were investigated in one subject by comparing the image that resulted from directly exciting the dissolved HP ^129^Xe magnetization (i.e., placing the excitation pulse 3826 Hz above the gas-phase resonance) to an image obtained by centering the excitation pulse 3826 Hz below the gas-phase resonance. The results of these control experiments are shown in Figure 3, which displays sections from a dissolved HP ^129^Xe image (3A) and the corresponding control image (3B).

The signal intensity generated by off-resonant ^129^Xe excitation was less than 10% of that generated by on-resonant excitation, and thus adds primarily to the diffuse background noise in the image. Indeed, similar noise is seen outside the lungs in Figure 2A. However, the spatial distribution of this off-resonance noise does not appear to be entirely random and could potentially contribute artifacts to the dissolved images. In future work, it should be possible to better suppress the gas-phase HP ^129^Xe signal with more carefully designed RF pulse shapes, elimination of RF leakage caused by un-blanking of the RF amplifier, and post-processing approaches.

### Postural Heterogeneity in Dissolved HP ^129^Xe MRI

**Figure 4:**
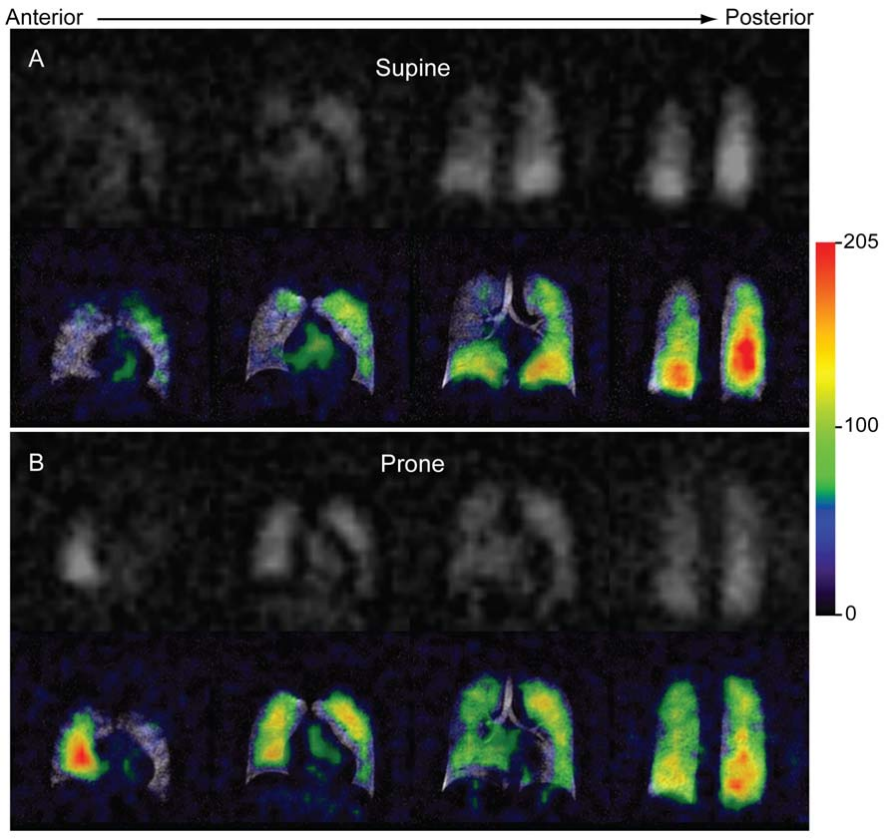
Postural dissolved HP ^129^Xe image heterogeneity. Dissolved ^129^Xe images displayed in grayscale (top) and overlaid in color (bottom) on the corresponding ventilation images. For the color overlays, the dissolved image signal intensity (arbitrary units) is indicated in the legend. The ventilation image was obtained in the supine position. (**A**) Dissolved image acquired after the subject had been supine for 1 hour. Note, the more gravitationally dependent, posterior portions of the lungs exhibited higher signal intensities than did the less dependent anterior regions. (**B**) Same subject imaged 10 minutes after moving to the prone position. Again, the gravitationally dependent (now anterior) regions display increased signal intensity.

Figure 4 displays dissolved HP ^129^Xe images from a single subject in grayscale and in color overlaid on the corresponding ventilation image. The dissolved ^129^Xe images largely match the normal ventilation patterns but display significant directional, heterogeneity. (Note, the strong appearance of iso-gravitational heterogeneity in these images is most likely the result of relatively low SNR.) When the subject was supine (Figure 4A), the signal intensity of the dissolved ^129^Xe MR image increased notably toward the posterior image sections. The dissolved ^129^Xe images from all other supine subjects displayed similar patterns. Conversely, when this subject was imaged in the prone position (Figure 4B), the HP ^129^Xe signal increased in the anterior portions of the image. That is, in both cases, higher signal intensity was observed from the more gravity-dependent portions of the lungs.

Gravity has long been known to affect both pulmonary perfusion and ventilation [30], and, as a result, subject position (i.e., prone versus supine) strongly influences these aspects of pulmonary function [31], [32], [33]. In the supine position, ventilation is known to decrease in the anterior and increase in the posterior portions of the lungs. Antero-posterior signal intensity gradients have also been noted in HP ^3^He MRI studies of human ventilation [34], [35]. These gradients presumably result from increased ventilation in the dependent portions of the lungs, which leads to higher ^3^He spin densities in these regions. Although not investigated in this work, ventilation gradients may similarly affect gas-phase HP ^129^Xe and, through diffusive exchange, induce corresponding gradients in the dissolved HP^129^Xe signal intensity.

Gravity also deforms the pulmonary microstructure and, as has been demonstrated through morphological measurements of canine lungs [36], produces smaller alveolar sizes in the dependent relative to the non-dependent portions of the lungs. Gravitationally dependent gradients in alveolar size have also been detected in humans [37] through *in vivo* imaging of the HP ^3^He apparent diffusion coefficient (ADC), which is a sensitive measure of pulmonary microstructure and thus of alveolar size. Smaller alveoli correspond to higher alveolar surface-to-volume ratios and should produce faster rates of HP ^129^Xe magnetization transfer into the pulmonary tissues within the dependent portions of the lungs. Therefore, a more rapid uptake of HP ^129^Xe magnetization may also contribute to the signal intensity pattern observed in Figure 4.

Additionally, the signal intensity of dissolved HP ^129^Xe depends on the local tissue density. Previous studies of supine subjects using ^1^H MRI [38] and positron emission tomography (PET) [39] have demonstrated that the pulmonary tissue density increases by ∼40% in going from nondependent (anterior) to dependent (posterior) portions of the lungs. Although it is still vigorously debated whether these density gradients arise from gravitationally induced tissue deformation or the redistribution of pulmonary perfusion [40], it is clear that either effect should influence the distribution of the dissolved HP ^129^Xe.

### Contribution of Blood Volume to Dissolved HP ^129^Xe Signal Intensity

To aid in interpreting the dissolved-phase ^129^Xe images, it is instructive to consider how much of the signal originates from ^129^Xe in the gas exchange membrane versus ^129^Xe in pulmonary blood. This can by calculated from ^129^Xe spectra because the peak at 218 ppm is uniquely attributable to ^129^Xe in red blood cells, and the size of this peak, relative to the membrane tissue/plasma peak (197 ppm), is determined by the relative ^129^Xe solubility and the pulmonary hematocrit. The RBC peak (see Figure 1B) corresponds to ∼30% of the total dissolved ^129^Xe signal intensity. In healthy individuals, the pulmonary hematocrit is approximately 90% that of peripheral blood [41] (typically 36–52% [42]). The Ostwald solubility of xenon in blood plasma and RBCs at 37°C is 0.0939 and 0.2710, respectively [43]. From these values, one can conclude that 40 to 50% of the total dissolved HP ^129^Xe signal originates from the blood.

As was previously shown in rats [17], [44], a portion of this blood-based signal could originate from dissolved HP ^129^Xe located beyond the gas-exchange tissues, for instance in the pulmonary veins. Weak signal was occasionally observed even from the left atrium of the heart (see second panel in Figure 4A). However, beyond the alveoli, dissolved HP ^129^Xe magnetization will be rapidly attenuated by RF and longitudinal relaxation [45], because there is no diffusive replenishment from the gas-phase reservoir. Therefore, during MR imaging, signal will be observed almost exclusively from the gas exchange tissues (i.e., the pulmonary capillary bed and associated parenchymal tissues).

Because a significant portion of the dissolved HP ^129^Xe signal originates from the blood, the blood volume within the pulmonary capillaries is expected to influence the signal intensity pattern observed in dissolved-phase MR images. Pulmonary capillaries are often assumed to be either open (i.e., recruited) and actively perfused or closed (non-recruited) and completely collapsed [46], [47]. This assumption suggests that the thickness of the alveolar septum in a perfused region should be ∼10 µm (2× the membrane thickness plus the RBC diameter [4]) and ∼2 µm (2× the membrane thickness) in non-perfused regions. However, studies of excised lungs employing micro-beads indicate that capillaries can retain functional diameters of ∼1.7 µm even under pressure conditions expected to completely collapse the capillary bed [48]. Therefore, perfusion by blood plasma might continue, even if RBCs do not enter the pulmonary capillaries. Still, the thickness, and therefore the volume, of plasma-only perfused alveolar septa would still be less than fully perfused (i.e., plasma and RBCs) septa and therefore exhibit lower ^129^Xe signal. By contrast, the fully perfused portions of the lungs will have larger septal volumes and, combined with the nearly threefold higher solubility of xenon in RBCs relative to blood plasma [43], should exhibit substantially higher ^129^Xe signal intensities.

### Diagnostic Potential of Dissolved HP ^129^Xe MRI

The sensitivity of the dissolved HP ^129^Xe signal to diffusive gas exchange and to various aspects of lung microstructure make HP ^129^Xe a promising contrast agent for studying and diagnosing pulmonary diseases. For instance, the dissolved ^129^Xe signal intensity will yield attenuated signal intensity from regions of the lungs that have undergone emphysematous tissue destruction. Additional insights could be gained by exploiting the chemical shift difference between HP ^129^Xe dissolved in the red blood cells (218 ppm resonance) and HP ^129^Xe dissolved in the blood plasma/parenchymal compartments of the pulmonary gas exchange tissues (197 ppm).

As was demonstrated previously in a rats [21], it is possible to use a 1-point, Dixon-based technique to differentially image ^129^Xe bound to the RBC versus ^129^Xe dissolved in the plasma/parenchymal compartments and thus, to detect regions of gas exchange impairment caused by pulmonary fibrosis. Similarly, images resulting from ^129^Xe within the RBCs should reveal regions of restricted capillary blood flow caused by pulmonary embolism. Thus, in combination with high-resolution ventilation images, dissolved HP ^129^Xe MRI could yield data akin to that obtained from scintigraphic ventilation/perfusion imaging [49], but with superior temporal resolution and without the need for ionizing radiation.

To be an effective diagnostic tool, however, image acquisition must be accomplished during an acceptably short breath-hold period. While the 16-second-breath-hold used in this work is similar to those used in HP ^3^He MRI studies of subjects with severe asthma [50] and COPD [51], it might be too long for some patients. However, the 3751 radial imaging views used in this work exceeds the number needed to fully sample k-space (3217 are required for a 32^3^ image). Thus, the total acquisition time could be reduced by ∼14% with only a trivial modification of the imaging strategy. Moreover, radial imaging is quite robust to artifacts caused by collecting undersampled data [23], [52]. This insensitivity to undersampling suggests that image acquisition times could easily be reduced by a factor of two or more, making dissolved ^129^Xe imaging possible in most patient populations. Similarly, it should enable the acquisition of higher-resolution (larger matrix) images with only minor increases in image acquisition time. Moreover, the resulting SNR reductions could be overcome using improved polarization technology, which can generate liter quantities of HP ^129^Xe at polarizations exceeding 50% [53].

To fully exploit the potential of dissolved ^129^Xe MR imaging, it will be necessary to develop metrics for quantifying the dissolved signal intensity. Specifically, it will be necessary to determine the relative signal intensity contributions from tissue density, regional ventilation, and HP ^129^Xe magnetization dynamics (i.e., the combination of ^129^Xe relaxation processes, diffusive exchange, and alveolar microstructure). While it is possible to calculate the magnetization dynamics with an appropriate diffusion-based model [16], [21], the other contributions will likely require additional information. Fortunately, as is done in arterial spin labeling studies of pulmonary perfusion [33], it should be possible to estimate the local tissue density using ^1^H MRI [54]. Proton MR images will also provide an anatomical reference for co-registering dissolved ^129^Xe images with HP ^129^Xe ventilation images, which may be necessary for quantitative comparisons. To alleviate the need for image registration, simultaneous ventilation and dissolved HP ^129^Xe images could be acquired using the chemical shift imaging approach recently proposed by Mugler *et. al.* [55]. Alternatively, it should be possible to image HP ^129^Xe in both phases within a single breath using an interleaved radial acquisition that alternates between the exciting the gas and the dissolved phases.

### Conclusions

We have demonstrated that it is possible to directly image HP ^129^Xe dissolved in human lungs within a single held breath. These 3D images display significant, directional heterogeneity, which is consistent with the known effects of gravity on ventilation, alveolar size, tissue density, and perfusion. Clearly, additional work is needed to quantify the influence of these morphological and physiological factors on signal intensity. However, our results suggest that dissolved ^129^Xe MRI holds the potential to yield analogous, but spatially resolved, information to that obtained through conventional DL_CO_ measurements and thus, provide a non-invasive means to detect regional impairment of gas exchange and to evaluate pulmonary diseases over time.
